# Extracellular Vesicles in Sepsis: Pathogenic Roles, Organ Damage, and Therapeutic Implications

**DOI:** 10.7150/ijms.86832

**Published:** 2023-10-16

**Authors:** Ni An, Zhe Chen, Peng Zhao, Wen Yin

**Affiliations:** 1 Department of Emergency, Xijing Hospital, Air Force Medical University, Xi'an, China.; 2 University College London, London, UK.

**Keywords:** sepsis, extracellular vesicles, inflammation, exosomes, organ dysfunction

## Abstract

Despite significant advances in anti-infective treatment and organ function support technology in recent years, the mortality rate of sepsis remains high. In addition to the high costs of sepsis treatment, the increasing consumption of medical resources also aggravates economic pressure and social burden. Extracellular vesicles (EVs) are membrane vesicles released from different types of activated or apoptotic cells to mediate intercellular communication, which can be detected in both human and animal body fluids. A growing body of researches suggest that EVs play an important role in the pathogenesis of sepsis. In this review, we summarize the predominant roles of EVs in various pathological processes during sepsis and its related organ dysfunction.

## Introduction

Sepsis is a syndrome of systemic inflammatory response caused by the invasion of pathogenic microorganisms such as bacteria into the organism. Every year, 50 million people suffer from sepsis and 11 million die as a result 1. Sepsis may cause shock, multiple organ failure, and even death if not detected or treated quickly. The Global Sepsis Alliance reports that the number of patients hospitalized for sepsis has doubled over the past 10 years 2. Sepsis is a high heterogeneous disease, and the heterogeneity of sepsis at the individual patient level has hindered progress in the field beyond current treatment standards. In addition, sepsis has a rapid onset, symptoms similar to other disorders, and no specialized tests, so rapid diagnosis and treatment of sepsis is essential. Most sepsis deaths can be avoided with rapid diagnosis and treatment. Early treatment of sepsis not only improves the patient prognosis, but also reduces the hospital stay, which is cost-effective and resource-conserving.

EVs are a kind of membranous small vesicles released by cells to the extracellular matrix, with a particle size distribution range from 30 nm to 1 μm, which play a key role in intercellular communication and body regulation through signaling molecules such as proteins and lipids on the membrane, as well as neurotransmitters, enzymes, hormones and nucleic acids wrapped in the membrane 3-5. The composition of EVs cargoes is complex, containing hundreds to thousands of different proteins, unique lipids, some DNA, and numerous small non-coding RNAs. There is evidence that EVs are involved in many physiopathological processes, including cellular homeostasis, infection transmission, cancer development, and cardiovascular disease 6-9. Over the past two decades, a large number of original studies investigating sepsis EVs have been published 10-13. In this review, we mainly introduce the changes of EVs cargoes in sepsis and EV functions in the sepsis-related organ dysfunction.

## Cargoes of EVs in sepsis

There is a positive correlation between the number of EVs and the severity of sepsis when sepsis is present or when bacteria irritate the body 14. EVs carrying altered proteins have been found in the fluids of patients with sepsis, which may contribute to the progression of the disease 15. Acute-phase reactive proteins and immunoglobulins, which are involved in the inflammatory response, are upregulated in the early stages of sepsis 16. A variety of cell types, including activated macrophages, monocytes, and neutrophils, generate EVs with altered protein profiles 17. In serum of sepsis mice, a number of cytokines and chemokines are specifically encapsulated in exosomes, and exosome inhibitor GW4869 can reduce exosome formation and inflammatory cytokine release significantly 18. There is evidence that cytokines and chemokines in exosomes are different from serum-free cytokines and chemokines in that they may have a role in lymphocyte differentiation and proliferation 19. Exosomes derived from macrophages stimulated by LPS also produced high levels of cytokines. According to recent studies, EVs released by different types of cells carry molecular patterns associated with damage, including high mobility group box 1 protein, histones, and extracellular cold-induced RNA-binding protein 19.

It is important for nucleic acid transport to use EVs, which protect nucleic acids from degradation by nucleases and keep them stable. In sepsis, EVs carry a variety of nucleic acids, including mRNA, microRNA, long noncoding RNA (lncRNA), and circRNA. In patients with sepsis, EVs express higher amounts of mRNA involved in antioxidant defense and oxidative stress 20. The microRNA expression profile of EVs is altered in sepsis and may be associated with the risk, severity, and prognosis of sepsis 21, 22. MicroRNAs play a role in sepsis via a variety of pathways, including immunomodulation, microvascular dysfunction, and organ dysfunction 5. In addition, lncRNA, and circRNA were also altered. Studies showed that EVs carrying the lncRNA NEAT1 in sepsis have been found to aggravate sepsis-related encephalopathy, and lncRNA-p21 could inhibit LPS-induced lung cells injury 23, 24. Serum exosomes from patients suffering from sepsis were up-regulated with hsa_circRNA_104484 and hsa_circRNA_104670, suggesting that they could be used as diagnostic markers for the disease 25.

## EVs and inflammation in sepsis

EVs originating from diverse cellular sources have been substantiated to exert significant roles in various biological processes. In the course of sepsis pathogenesis, pathogens (such as bacteria, viruses, or fungi) or their toxins induce systemic infection 26. These pathogens or their toxins stimulate the immune system, triggering the generation of abundant inflammatory cells and mediators, which disseminate throughout the body via the circulatory system, consequently provoking Systemic Inflammatory Response Syndrome (SIRS) and leading to organ failure and death 27, 28.

Gram-positive and Gram-negative bacteria, as the most prevalent infectious agents in sepsis, can produce EVs carrying bacterial endotoxins and transmitting bacterial proteins 29, which enter the septic patient's fluid circulation 30. During Gram-negative bacterial infection, outer membrane vesicles (OMVs) serve as crucial facilitators for the entry of LPS and caspase-11 activation into the cytoplasm 31. OMVs bearing specific antigens, dependent on TLR2 or TLR4, induce activation of B cells and CD4(+) T cells, resulting in the activation of adaptive immunity 32, 33. Concurrently, through various pathways, including the NF-κB signaling cascade, innate and adaptive immune responses in sepsis are activated 34, thereby inducing systemic inflammation 35-37.

During sepsis, host-derived EVs are predominantly produced by immune cells, such as platelets and innate immune cells 3. It is generally believed that EVs originating from immune cells exacerbate the onset of sepsis, as they carry higher levels of damage-associated molecular patterns (DAMPs) and cytokines 38. They can activate various pattern recognition receptors (PRRs) and signaling pathways to induce pro-inflammatory responses 39, 40, such as the release of pro-inflammatory cytokines IL-12, IL-15, IL-17, and IFN-γ, promoting macrophage proliferation and M1 polarization, ultimately leading to a "cytokine storm" 41, 42 **(Table 1)**. In addition to activating innate immune responses, EVs in sepsis also induce Th1/Th2 cell differentiation, enhance T lymphocyte proliferation and migration during the course of sepsis, and activate adaptive immune responses 41, further mediating inflammation. Inhibition of the exosome generation process has been shown to reduce the inflammatory response and significantly improve survival rates in sepsis 43. Recent research has demonstrated that modifying miRNA within extracellularly generated exosomes can suppress the cytokine storm in sepsis and inhibit its development 44. Interestingly, some studies have shown that certain EVs may suppress inflammation in septic patients, for example, EVs containing α-2-macroglobulin secreted by neutrophils contribute to bacterial clearance and alleviate inflammation 45. Moreover, immature dendritic cell-derived EVs mitigate acute systemic inflammatory responses in sepsis by enhancing apoptotic cell clearance 46. Overall, EVs can balance pro-inflammatory responses and immune suppression.

However, there is a scarcity of research on the role of EVs in the immunosuppressive mechanisms of sepsis. Unveiling their functional mechanisms will provide insights for immune-based therapeutic approaches for sepsis.

## EVs and cardiovascular function in sepsis

A close relationship exists between sepsis and cardiovascular dysfunction. Arterial hypotension is the most common feature of cardiovascular dysfunction in septic patients, primarily due to factors such as reduced blood volume, decreased vascular tone, and myocardial suppression 62. These factors lead to a decline in left and right ventricular ejection fractions, potentially causing severe consequences. Patients with sepsis-associated cardiovascular dysfunction often exhibit poor tolerance to fluid administration, which is related to decreased central venous oxygen saturation (ScvO2) 63, 64. Adequate fluid resuscitation to increase blood volume and improve microcirculation is crucial for maintaining tissue oxygenation; however, achieving this goal may be difficult for septic patients due to cardiovascular dysfunction.

EVs may play a critical role in cardiovascular dysfunction during sepsis. *In vitro* experiments have demonstrated that OMVs from a uropathogenic Escherichia coli strain induce irregular calcium oscillations with reduced frequency in cardiomyocytes. Following intraperitoneal injection of sterile OMVs, OMVs can be detected within the heart. Troponin T levels in the blood significantly increase, while echocardiography reveals increased heart wall thickness and heart rate 65.

During the progression of sepsis, the assembly of the TXNIP-NLRP3 complex in monocytes can be embedded into CD63+ exosomes, transported from circulating monocytes to local macrophages, and promote the cleavage of inactive IL-1β and IL-18 in macrophages, exacerbating cardiovascular inflammation and myocardial dysfunction 66. Moreover, hsa-miR-1262 in sepsis-derived EVs may interact with SLC2A1, thereby inhibiting glycolytic activity and promoting cardiomyocyte apoptosis 67. Platelet-derived exosomes from septic patients generate nitric oxide (NO), which contributes to sepsis-induced myocardial dysfunction and vascular damage 68. One study demonstrated that exosomes from septic patients induced endothelial cell and apoptotic cell vascular smooth muscle, a result of increased exosomal NADPH activity 69. Likewise, another study showed that platelet-derived exosome production in sepsis may be regulated by NO and bacterial components, promoting the generation of reactive oxygen species, peroxynitrite, caspase-3 activation, and vascular endothelial cell apoptosis, ultimately causing vascular dysfunction in sepsis 70.

## EVs and coagulation dysfunction in sepsis

Most sepsis patients exhibit coagulation dysfunction, which varies in severity. The most severe coagulation disorder in sepsis is disseminated intravascular coagulation (DIC), characterized by systemic thrombus formation and bleeding 71. During the development of sepsis, EVs display procoagulant tissue factors and phosphatidylserine on their surfaces, thereby modulating inflammatory responses and coagulation 72. Tissue factor is a cell surface receptor for coagulation factors VII/VIIIa and plays a crucial role in initiating the extrinsic coagulation pathway. Escherichia coli OMVs induce coagulation in a TLR4-dependent manner 73, and increase TF activity within the organism via the caspase-11-GSDMD pathway 74, further promoting a hypercoagulable state in sepsis 75. Under normal physiological conditions, tissue factor is not expressed within cells; however, during bacterial infections, tissue factor appears in the blood to inhibit bacterial dissemination and limit the impact of microbes on the host 76, 77. Nevertheless, excessive coagulation activation can lead to impaired tissue circulation, endothelial dysfunction, and organ damage 62, 78.

Increased circulating EVs derived from endothelial cells, platelets, red blood cells, and white blood cells are associated with coagulation activation, tissue factor release, inflammation, and ROS production, leading to small intravascular thrombi and endothelial damage 79-81. Studies have shown that EVs isolated from plasma during sepsis impair erythrocyte deformability 82. As tissue factor is primarily expressed by activated monocytes in the body, EVs transporting tissue factor are considered to be mainly released from activated monocytes 83, forming tissue factor- and CD13-positive EVs that promote inflammation and coagulation. The proportion of tissue factor- and CD13-positive EVs increases with symptom severity. Other studies have also demonstrated a strong positive correlation between procoagulant tissue factor activity in EVs and the severity of sepsis 84, and reported that the content of procoagulant tissue factor in circulating EVs is associated with the onset of DIC 77, and the formation of microthrombi, activation of the coagulation and fibrinolytic systems, and activation of the complement system occur, thereby activating white blood cells and endothelial cells to promote the release of pro-inflammatory mediators, further intensifying the onset of inflammation 85, 86, and further leading to coagulation dysfunction. Additionally, platelet-, leukocyte-, and endothelial cell-derived EVs containing phosphatidylserine on their surfaces can promote coagulation activity during sepsis; however, their procoagulant roles in sepsis remain unclear 87, 88. Leukocyte-derived EVs inhibit endothelial nitric oxide synthase activation, enhance inducible nitric oxide synthase (iNOS) expression *in vivo*, induce systemic vasodilation, and reduce mean arterial pressure in septic infectious shock 89.

## EVs and acute lung injury in sepsis

Sepsis frequently leads to pulmonary inflammation, further progressing to acute lung injury (ALI) and acute respiratory distress syndrome (ARDS) 90, causing irreversible lung damage primarily characterized by diffuse alveolar damage, hypoxemia, and respiratory distress 91. Studies suggest that lung injury may stem from direct lung injury caused by epithelial damage and indirect lung injury induced by endothelial cell damage 92, 93. During the development of sepsis-induced lung injury, cytokines mediate the aggregation and infiltration of numerous immune cells in lung tissue, activating a positive feedback loop for the inflammatory response, ultimately culminating in a cytokine storm and disrupting the alveolar-capillary endothelial barrier structure, allowing neutrophils and macrophages to infiltrate the alveoli 94, 95.

In sepsis-induced ALI and ARDS, both bronchoalveolar lavage fluid (BALF) and circulating EVs exhibit upregulation in quantity 96, 97. EVs within BALF are predominantly secreted by alveolar macrophages 98, and the activation of alveolar macrophages, as initiators of innate immune system activation, can induce inflammatory responses. Concurrently, research reveals that damaged pulmonary epithelial and endothelial cells release EVs containing various miRNAs (miR-221 and miR-320a), cytokines, and caspase-3, potentially activating alveolar macrophages to release multiple pro-inflammatory mediators 99-102, promoting macrophage EV release and stimulating TNF-α expression 103. Similarly, following LPS injection in mice, alveolar macrophage EV release rapidly increases within one hour 104. Furthermore, EVs released by activated alveolar macrophages can activate and recruit resting macrophages, subsequently activating the NLRP3 inflammasome and intensifying the inflammatory response caused by sepsis 105, 106. In addition to EVs derived from alveolar macrophages, monocyte EVs may induce pulmonary endothelial cell damage and mediate pyroptosis through cleaved GSDMD and active caspase 1 107, 108, while endothelial cell damage and the release of nitrated S1PR3-containing EVs could exacerbate pulmonary inflammation 109. Moreover, various circulating cell-derived EV contents, including miR-145, miR-210-3p, and miR1-3p, can impact pulmonary inflammation 110, 111 and endothelial barrier dysfunction 112 via different cellular signaling pathways, although the source of these plasma EVs requires further investigation.

The viral infection caused by SARS-CoV-2 can lead to symptoms similar to those of sepsis caused by bacterial infections. 113. Similar to bacterial sepsis, SARS-CoV-2 infection can cause pulmonary inflammation, alveolar damage, gas exchange impairment, and shock 114, although the distinction lies in SARS-CoV-2 directly invading the lungs to cause injury. Several inflammatory factors, such as IL-6, TNFα, IL-1β, and granulocyte-colony stimulating factor, may play pivotal roles in acute lung injury caused by SARS-CoV-2 115-117. Research indicates that EVs exhibit elevated immune and vascular-related markers in patients with moderate-to-severe SARS-CoV-2 infection 118. Additionally, pulmonary EVs may carry ACE2, allowing SARS-CoV-2's spike protein to enter target cells via binding to the ACE2 on EVs 119, thereby playing a role in the pathogenesis of the disease.

## EVs and acute kidney injury (AKI) in sepsis

In septic patients, renal dysfunction is a common and severe complication. Elevated serum urea (or blood urea nitrogen) and creatinine are common in sepsis-associated renal dysfunction, and even mild increases in creatinine concentration are associated with poorer prognosis in critically ill patients 120, 121. Acute kidney injury (AKI) is a serious renal dysfunction, clinically characterized by oliguria (reduced urine output), usually secondary to sepsis-induced infectious shock and hypovolemia 122.

EVs from various cellular sources may have regulatory effects on sepsis-mediated acute kidney injury, possibly related to the multiple non-coding RNAs carried by the EVs **(Table 2)**. The increased number of platelet-derived EVs is negatively correlated with AKI biomarkers blood urea nitrogen and creatinine concentrations 123. Adipose-derived mesenchymal stem cell (MSC)-derived EVs significantly suppress renal oxidative stress and inflammatory response 124, and further research suggests that the protective effect of adipose-derived MSC-derived EVs may be mediated through the SIRT1 signaling pathway 125. Another study demonstrated that exogenous umbilical cord MSC-derived EVs can inhibit the NF-κB signaling pathway and attenuate renal inflammatory infiltration through miR-146b 126. EVs derived from endothelial progenitor cells may, through their encapsulated miR-382-3p, target the E3 ubiquitin-protein ligase (BTRC), thereby ameliorating the IκBα/NF-κB axis and inhibiting immune responses in multiple organs, including the kidney 126. Additionally, studies revealed that endothelial progenitor cell-derived EVs deliver miR-21 to regulate RUNX1, thereby reducing oxidative stress, inflammation, and apoptosis levels in renal tubular epithelial cells 127. Multiple studies have shown that non-coding RNAs in EVs released by renal tubular epithelial cells during AKI may mediate macrophage polarization towards a pro-inflammatory M1 phenotype 128-130. The polarization state of macrophages may also affect the effects of their released EVs on renal tubular epithelial cells; M1 macrophage-derived EVs promote renal epithelial cell apoptosis, while M2 macrophage-derived EVs carry miR-93-5p, which suppresses renal epithelial cell pyroptosis and alleviates AKI by regulating TXNIP 131. Therefore, the communication and dialogue between macrophages and renal tubules through EVs may be a critical factor in acute kidney injury in sepsis. Interestingly, in sepsis induced by CLP, Limb myotubes might exert remote ischemic preconditioning on the kidney due to hypoxia. Through HIF-1α dependent upregulation of miR-21, by targeting PDCD4/NF-κB and PTEN/AKT pathways in renal tubular epithelial cells, thereby exerting anti-inflammatory and anti-apoptotic effects 132. However, in addition to the direct regulatory effects on renal cells, EVs-induced coagulation dysfunction in sepsis also promotes thrombus formation in the renal microcirculation 3, 133, leading to disordered intrarenal perfusion and medullary hypoxia 134.

## EVs and neurological dysfunction in sepsis

The impact of sepsis on the brain primarily manifests as acute and long-term neurological dysfunction, including sepsis-associated encephalopathy and cognitive impairment 136. The pathogenesis mainly involves the interplay of systemic inflammation, blood-brain barrier dysfunction, neuroinflammation, microcirculatory dysfunction, and cerebral dysfunction 137, 138. Following LPS stimulation, choroid plexus epithelial cells secrete EVs containing inflammatory proteins and miRNAs (miR-146a and miR-155), which are absorbed by astrocytes and microglia via cerebrospinal fluid and transmit inflammatory information, thereby affecting the central nervous system 139.

In the cecal ligation and puncture (CLP) rat model, blood-brain barrier damage and increased reactive oxygen species (ROS) levels promote ferroptosis, during which the expression of serum exosomal NEAT1 is upregulated, potentially regulating miR-9-5p/TFRC and GOT1 axis through a competing endogenous RNA mechanism to promote neuronal ferroptosis in rats, thus exacerbating sepsis-associated encephalopathy 140.

## EVs and liver injury in sepsis

Although there are no overt structural abnormalities in the liver and biliary system in septic patients, alterations in liver function remain common. Liver dysfunction is mainly characterized by elevated bilirubin or transaminase levels 141. Liver injury may lead to changes in the clearance rate of bacteria or LPS and result in the release of pro-inflammatory cytokines, further exacerbating the symptoms of sepsis 142. Studies have found that macrophages release high-mobility group box 1 (HMGB1) via EVs as a DAMP, mediating cytotoxicity and leading to cell death and tissue injury. The interaction between HMGB1 and the receptor for advanced glycation end-products is involved in loading HMGB1 into EVs. Through transferrin-mediated endocytosis, these EVs transfer HMGB1 to target cells, activating the NLRP3 inflammasome, and consequently resulting in hepatocyte pyroptosis 143, 144.

## MCS-derived EVs, the other side?

While EVs derived from bacteria and immune cells may precipitate various complications in sepsis, those originating from mesenchymal stem cells (MSCs) have been found to play a pivotal role in different experimental models of acute tissue injury during the process of sepsis, which are largely attributed to the paracrine actions of MSC-EVs. These exosomes can either interact with receptors on the surface of target cells or fuse with them, releasing their contents into the cell and subsequently altering the function of the recipient cell 145.

In cardiovascular system, MSC-derived EVs have been identified that may contribute to this regulatory effect, including carrying PTEN-induced putative kinase 1, which ameliorates mitochondrial dysfunction in cardiomyocytes 146. Additionally, several MSC-carried miRNAs, such as miR-233 147 and miRNA-141 148, may be involved in cardioprotective effects. In the kidneys, MSC-derived EVs have been shown to provide renal protection by inhibiting oxidative stress, cell apoptosis, and fibrosis, and promoting autophagy 127, 149. They also achieve immune regulation by inducing the anti-inflammatory and immunosuppressive effects of M2 macrophages and regulatory Tregs 150, and by modulating NK cells 151, 152. This mechanism may also be attributed to the broad gene regulatory effects of their miRNA contents, as evidenced by a wealth of preclinical studies 127, 149, 153, 154**.** Similarly, the administration of MSC-derived EVs to patients with acute lung injury caused by sepsis in clinical settings has shown comparable protective effects. Intravenous injection of MSC-derived EVs can increase the rate of alveolar fluid clearance, reduce pulmonary protein permeability, enhance antibacterial activity 155, and significantly improve acute lung injury patient's survival rates caused by COVID-19 and Epidemic Influenza A 156, 157. Nevertheless, further confirmation is needed to validate the protective role of MSC-derived exosomes on different organs in sepsis. In addition, despite the demonstrated protective effects of MSC-derived EVs on various tissue injuries, the effectiveness and consistency of MSC-derived EVs functions still require further evaluation for clinical treatment.

## Conclusions

**Figure 1:**
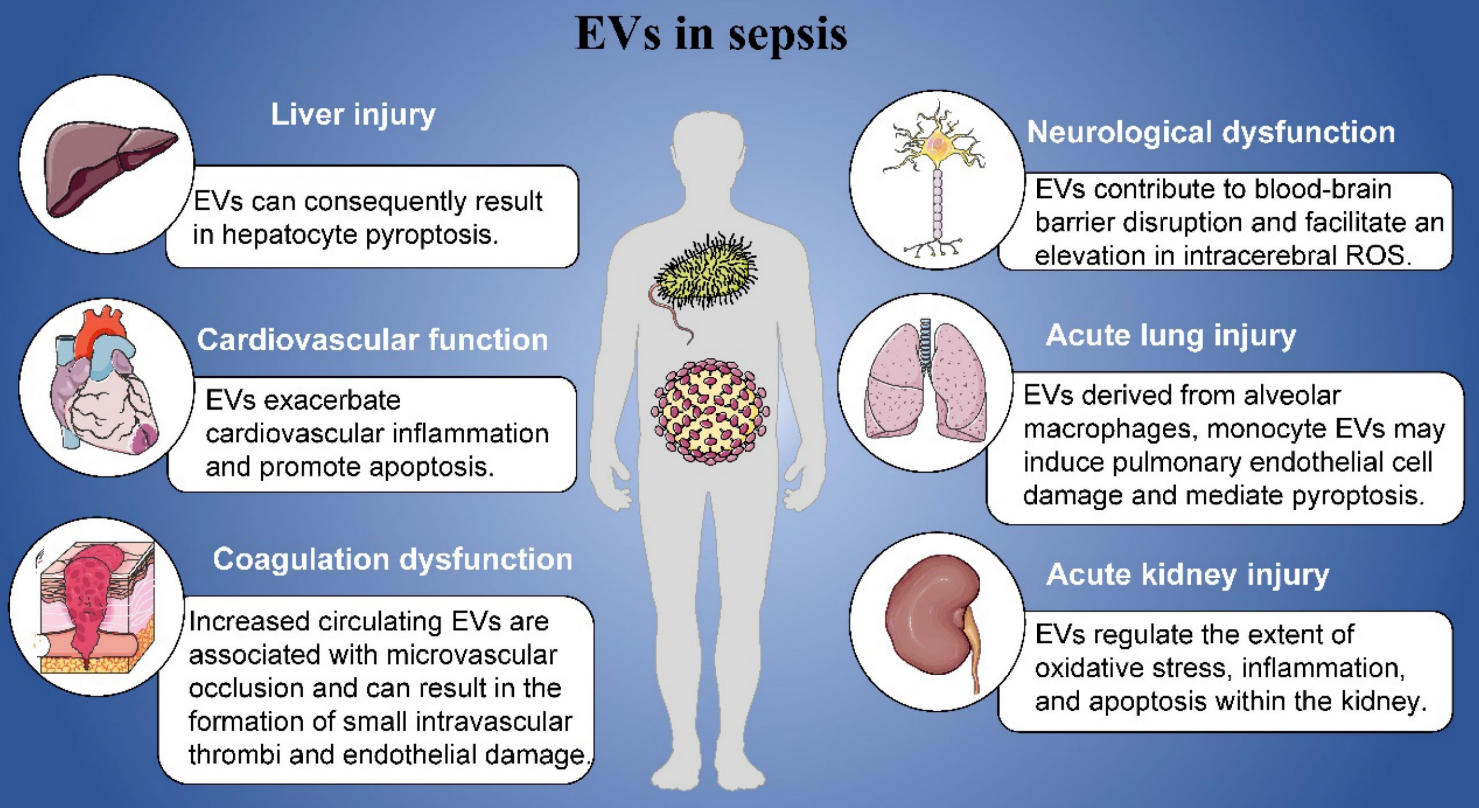
Role of EVs in sepsis.

In this review, we mainly introduced the roles of EVs in the pathogenesis of sepsis including cardiovascular function, coagulation dysfunction, acute lung injury, acute kidney injury, neurological dysfunction and liver injury **(Figure 1)**. Sepsis manifests as a pleiotropic process in which exosomes carry a multitude of proinflammatory molecules, activate cellular signaling, and cause multiple organ malfunctions. Based on the contents of EVs, EVs might play specific roles in the organ damages and other complications induced by sepsis. Furthermore, we have detailed the mechanisms by which EVs exert their influence in this process. At the same time, it has also been pointed out that EVs derived from mesenchymal stem cells may play a positive effect in different organ of patients with sepsis, that is, they can significantly inhibit acute organ injury caused by sepsis. Studying its regulatory mechanism in sepsis can provide a theoretical basis for future diagnosis, treatment strategies, and vaccine prevention.

## Figures and Tables

**Table 1:** The cytokines within exosomes in sepsis participate in immune regulation and peak timing 19

Cytokines	Function	Mechanism	Peak time in EVs	Peak time in Serum
IL-1β^47^, ^48^	Pro-inflammatory	Induces inflammation and stimulates immune responses.	12h	24-48h
IL-2^49^	Pro-inflammatory	Stimulates T-cell proliferation and differentiation.	2-12h	12h
IL-6^50^	Pro-inflammatory	Induces acute phase response and B-cell differentiation.	2h	2-12h
IL-12^51^	Pro-inflammatory	Promotes Th1 cell differentiation, stimulates NK cells.	24h	12-24h
IL-15^52^	Pro-inflammatory	Supports NK cell survival, stimulates T-cell proliferation.	2h	12h
IL-17^53^	Pro-inflammatory	Induces pro-inflammatory responses, promotes neutrophil recruitment.	24h	12h
TNF-α^48^	Pro-inflammatory	Regulates immune cells, induces inflammatory response.	2h	2h
IFN-γ^54^	Pro-inflammatory	Activates macrophages, promotes Th1 cell differentiation.	12-24h	12h
IL-4^55^	Anti-inflammatory	Promotes Th2 cell differentiation, B-cell activation.	24h	24h
IL-5^55^	Anti-inflammatory	Induces eosinophil activation and differentiation.	24h	12h
IL-10^55^	Anti-inflammatory	Inhibits inflammatory cytokines, regulates immune response.	24h	2-12h
CCL2^56^	Chemotactic factor	Chemotactic for monocytes, memory T cells.	12h	2-12h
CCL3^57^	Chemotactic factor	Chemotactic for NK cells, monocytes, dendritic cells.	12h	2h
CCL5^58^, ^59^	Chemotactic factor	Chemotactic for T cells, eosinophils, basophils.	24h	12h
CXCL9^60^, ^61^	Chemotactic factor	Chemotactic for T cells, promotes Th1 response.	24h	12h
CXCL10^60^, ^61^	Chemotactic factor	Chemotactic for T cells, NK cells, promotes Th1 response.	24h	12h

**Table 2:** The Role of Non-Coding RNAs Carried by EVs in Sepsis-Induced Acute Kidney Injury

miRNA	Sources	Function	Mechanism
miR-146b^126^	mesenchymal stem cell	Attenuation of renal inflammatory infiltration	Inhibition of the NF-kB Signaling Pathway
miR-93-5p^131^	M2 Macrophages	Inhibition of renal epithelial cell pyroptosis	Inhibition of the TXNIP-NLRP3 axis
miR-21^127^, ^132^	Limb myotubes, Endothelial Progenitor Cells	Inhibition of renal epithelial cell Inflammation and Apoptosis	Inhibition of the RUNX1, PDCD4/NF-κB and PTEN/AKT Signaling Pathway
miR-382-3p^135^	Endothelial Progenitor Cells	Attenuation of renal inflammation	Inhibition of BTRC to restrain NF-kB Signaling Pathway
